# Comparison of Velocity-Based Performance and Velocity Loss Between Traditional and Safety Squat Barbells During the Squat Exercise

**DOI:** 10.3390/sports14040146

**Published:** 2026-04-09

**Authors:** Miguel Alarcón-Rivera, Leonardo Poblete-Sánchez, Cristian Salazar-Orellana, Eduardo Guzmán-Muñoz, Exal Garcia-Carrillo, Pablo Luna-Villouta, Héctor Fuentes-Barría

**Affiliations:** 1 Escuela de Ciencias del Deporte y Actividad Física, Facultad de Salud, Universidad Santo Tomás, Talca 3460000, Chile; mrivera3@santotomas.cl (M.A.-R.); leopobletee5@gmail.com (L.P.-S.); 2 Escuela de Kinesiología, Facultad de Salud, Universidad Santo Tomás, Talca 3460000, Chile; eguzmanm@santotomas.cl; 3 Escuela de Pedagogía en Educación Física, Facultad de Educación, Universidad Autónoma de Chile, Talca 3460000, Chile; 4 Department of Physical Activity Sciences, Faculty of Education Sciences, Universidad Católica del Maule, Talca 3480112, Chile; exal.garcia@gmail.com; 5 Department of Physical Activity Sciences, Universidad de Los Lagos, Osorno 5290000, Chile; 6 Departamento de Educación Física, Facultad de Educación, Universidad de Concepción, Concepción 4070371, Chile; pabloluna@udec.cl; 7 Vicerrectoría de Investigación e Innovación, Universidad Arturo Prat, Iquique 1110939, Chile

**Keywords:** assessment, athletes, muscle strength, resistance training, task performance and analysis, exercise test, velocity based training, weightlifting

## Abstract

The purpose of this study was to examine differences between squat variations performed with a traditional barbell (TRAD) and a safety squat bar (SSB) in university athletes, focusing on mean propulsive velocity (MPV), peak velocity (PV), and velocity loss (VL). Nineteen university athletes participated in a randomized crossover repeated-measures design. Participants performed squat exercises with both barbell types at 65% and 85% of one-repetition maximum (1RM) across multiple testing sessions. Neuromuscular performance indicators were assessed using a linear velocity transducer. Two-way repeated-measures ANOVA revealed significant main effects of barbell type and load for MPV and PV (all *p* < 0.05). Higher MPV values were observed with the SSB at both loading intensities, whereas higher PV values were observed only at 85% 1RM. For VL, a significant main effect of barbell type was found (*p* = 0.013), with no significant effect of load (*p* = 0.155) or interaction (*p* = 0.507). In conclusion, the SSB elicited higher movement velocities compared with the traditional barbell. These findings suggest that barbell selection may influence velocity-based performance outcomes during squat exercise. However, due to the cross-sectional design, these results should be considered preliminary.

## 1. Introduction

The pursuit of optimal athletic performance drives continuous exploration of strategies to enhance preparation, manage fatigue, and reduce injury risk [1]. Among the training methodologies developed in recent years, velocity-based training (VBT) has emerged as a valuable approach for prescribing and monitoring resistance training. VBT employs technology to measure barbell velocity and key mechanical outputs such as power, force, and velocity loss during exercise execution [2,3]. This approach provides a more individualized and dynamic alternative to traditional percentage-based methods, allowing practitioners to adjust loads in real time based on athletes’ daily readiness [3,4]. Importantly, VBT enables the prediction of one-repetition maximum (1RM) without the need for maximal testing, which is known to fluctuate due to internal (e.g., recovery, nutrition) and external (e.g., environment, temperature) factors [5,6].

Another critical advantage of VBT lies in its ability to monitor fatigue. Velocity loss (VL) during sets has been proposed as a reliable marker of neuromuscular fatigue and a useful tool for regulating training volume and intensity [7,8]. Traditional strength training regimens, when poorly monitored, can lead to excessive fatigue and impaired performance. In this context, the squat is widely recognized as a fundamental multi-joint exercise with strong transfer to sport-specific actions such as vertical and horizontal jumping and sprinting [9,10]. Recent evidence in university athletes has shown that mean propulsive velocity (MPV) correlates significantly with jump performance, reinforcing the relevance of velocity-based measures as indicators of neuromuscular performance [11]. This highlights the practical utility of velocity monitoring in strength training programs, particularly in athletic populations.

Within resistance training practice, the traditional back squat (TRAD) remains one of the most frequently prescribed exercises, alongside the front squat, to develop lower-body strength and power. More recently, the safety squat bar (SSB) has gained attention as an alternative, offering potential biomechanical and practical advantages. The design of the SSB allows for a more upright torso position, reduced trunk tilt and hip flexion, and greater activation of the lower trapezius, while simultaneously lowering demands on shoulder mobility compared with TRAD [12,13]. Complementary biomechanical work also shows that the SSB modifies trunk and hip mechanics compared with a standard barbell, and that bar placement influences kinematic and kinetic patterns around the sticking region [14,15]. These characteristics suggest that the SSB may provide a safer and more accessible option for athletes with shoulder or wrist limitations, while also potentially influencing mechanical outputs such as velocity and force [14,16].

Despite these biomechanical benefits, evidence on the performance effects of the SSB remains scarce. Most research to date has focused on biomechanical or electromyographic differences rather than direct comparisons of neuromuscular performance variables. Vantrease et al. [15] conducted the only study directly comparing TRAD and SSB, analyzing bar velocity and muscle activation at 65% and 85% of 1RM in trained men. The authors reported no significant differences between squat variations, although a non-significant decline in peak power across repetitions was noted. More recently, Staheli et al. [16], identified significant differences in MPV when comparing TRAD and SSB, suggesting that bar type may influence performance outcomes depending on the protocol. However, further research is needed to address inconsistencies across studies and to extend comparisons to velocity-based outcomes and fatigue-related measures, such as velocity loss.

Given the limited and inconclusive evidence, further investigation into the comparative effects of traditional and safety squat barbells on velocity-based performance is warranted. Based on previous biomechanical and velocity-based evidence, it was hypothesized that the safety squat bar would elicit higher mean propulsive velocity and peak velocity compared with the traditional barbell. Given the limited and inconsistent evidence regarding fatigue-related outcomes, differences in velocity loss were examined in an exploratory manner.

Therefore, the purpose of the study was to examine differences between these squat variations in university athletes with respect to the neuromuscular indicators (mean propulsive velocity (MPV), peak velocity (PV), and velocity loss (VL)) during the squat exercise. This analysis aims to provide new insights into the potential advantages of each barbell type and deliver practical recommendations to optimize strength training prescription and monitoring in athletic populations.

## 2. Materials and Methods

### 2.1. Study Design and Sample Size

This study employs a randomized crossover repeated-measures design [17]. The sample size calculation for this study was performed a priori using G*Power software (version 3.1.9.7). The design corresponded to a two-way repeated-measures ANOVA (2 × 2), in which the same participants completed the four testing conditions. The effect size (partial eta-squared, ηp^2^) was estimated from a previous study that compared knee extensor moment between two different bars [13]. An ηp^2^ of 0.20 was considered the minimum relevant effect size. Based on these parameters, with a significance level of α = 0.05 and a statistical power of 0.80, the analysis indicated that a total of 16 participants were required to achieve the desired power. Finally, 19 participants were recruited, exceeding the minimum calculated sample size and thereby ensuring greater statistical power to detect significant effects. 

### 2.2. Participants

A total of 19 university athletes (age: 22.81 ± 2.82 years; height: 175.07 ± 5.12 cm; weight: 82.07 ± 10.98 kg; BMI: 26.89 ± 3.97) voluntarily participated in this study. Before data collection, all participants signed an informed consent form approved by the ethics committee of the Universidad Autónoma de Chile (code 26-23, date 19 April 2023), and the study was conducted in accordance with the principles of the Declaration of Helsinki [18]. All participants were regularly engaged in structured strength training as part of their university sport participation and had at least six months of prior resistance training experience. The inclusion criteria were as follows: (a) affiliation with a university sports branch (soccer, basketball, volleyball, athletics, combat sports), and (b) a minimum of six months of experience in strength training. The exclusion criteria included the following: (a) injury or trauma that would hinder proper execution technique, (b) a BMI classified as obese (BMI > 30), (c) individuals with no experience in strength training, and (d) any medical condition that would preclude them from undergoing maximum loads. Participants were asked about their prior experience with SSB, and none reported having used it before.

### 2.3. One Repetition Maximum Testing

The 1RM testing protocol was implemented in accordance with the methodological recommendations of Grgic et al. [19]; the athletes began with a general warm-up consisting of 3–5 min of treadmill running, followed by dynamic stretching and mobility exercises for the ankles, hips, and shoulders. Afterward, the specific warm-up started with 10 repetitions using an unloaded barbell. A subsequent set was performed with a load 10–20% heavier, for 3–5 repetitions, followed by 2–3 min of rest. The load was then progressively increased by 10–20% until approaching the participant’s estimated maximum. At this point, athletes attempted a single repetition at the target load. If the attempt was successful, the weight was increased by 5–10% for the next trial. If the attempt failed, the load was reduced by 5–10% and reattempted. The highest successfully lifted load was recorded as the participant’s 1RM.

### 2.4. Neuromuscular Performance Indicators

Neuromuscular performance was assessed using velocity-derived indicators obtained during resistance exercises performed at 65% and 85% of 1RM. Mean propulsive velocity (MPV), peak velocity (PV), and velocity loss (VL) were assessed using a linear velocity transducer (Chronojump BoscoSystem, Barcelona, Spain). MPV was defined as the mean propulsive velocity averaged across all repetitions performed within each set. PV was defined as the highest instantaneous velocity reached during the concentric phase of each repetition and subsequently averaged across all repetitions within the set. VL was expressed as the percentage decline in velocity from the fastest repetition to the final repetition of each set. The device was attached to the right side of the barbell at its widest point, with the base magnetically fixed to a 25-lb steel plate placed on the floor. During squat execution, the transducer cable was aligned perpendicularly to the floor and directly beneath the bar, as previously described [20]. Data were recorded using Chronojump^®^ software (2.46 version) and subsequently exported to Microsoft Excel (16.89.1 version) for further analysis.

### 2.5. Warm-Up Protocol

Each session began with a standardized warm-up consisting of 3–5 min of treadmill running, dynamic stretching targeting the lower limbs (ankle dorsiflexion, hip flexion–extension, and hip rotations), and three sets of front planks lasting 30 s each. Participants then performed two sets of 10 repetitions with the unloaded bar, followed by one set of five repetitions at ~60% of the intended working load, one set of three repetitions at ~70%, and one set of two repetitions at ~80%. This progression was designed according to established recommendations for resistance training warm-up sets [21,22], to minimize unnecessary fatigue and ensure adequate neuromuscular readiness before the main sets.

### 2.6. Experimental Design

**Figure 1:**
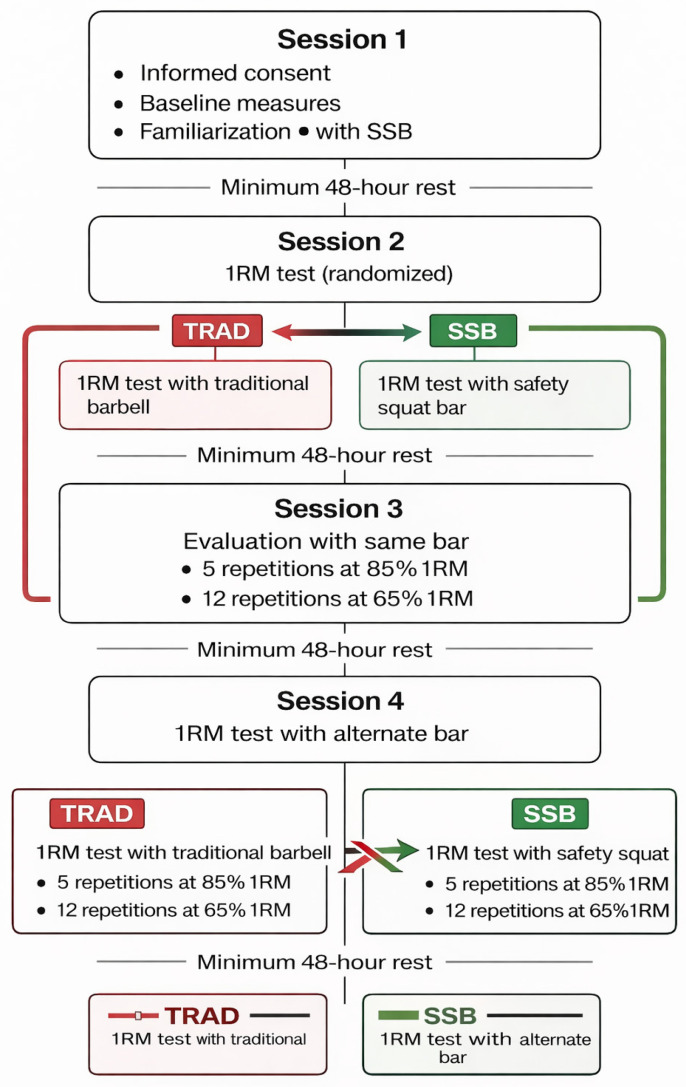
Experimental design and testing protocol. Participants completed five non-consecutive sessions over a two-week period using a randomized crossover design comparing the traditional barbell (TRAD) and the safety squat bar (SSB) at 85% and 65% of 1RM. Red boxes/text indicate the TRAD condition, green boxes/text indicate the SSB condition, and arrows indicate the crossover sequence between conditions. Bold text highlights the main testing or training components performed in each session.

The study employed a randomized crossover repeated-measures design to compare the safety squat bar (SSB) and traditional barbell (TRAD) at 85% and 65% of 1RM, as illustrated in Figure 1. Participants were already familiar with the traditional barbell squat through their regular resistance training practice. Therefore, the familiarization session was specifically conducted for the safety squat bar, as none of the participants had previously used this implement.

Testing was conducted across five non-consecutive sessions within a two-week period, each separated by at least 48 h to minimize fatigue and ensure recovery. The order of barbell conditions (SSB and TRAD) was randomized across participants to minimize potential order effects. During all testing sets, participants were instructed to perform each repetition with maximal intended concentric velocity. Repetitions were not performed to failure; instead, all sets were completed using a fixed number of repetitions as defined in the experimental protocol (five repetitions at 85% 1RM and twelve repetitions at 65% 1RM).

**Figure 2:**
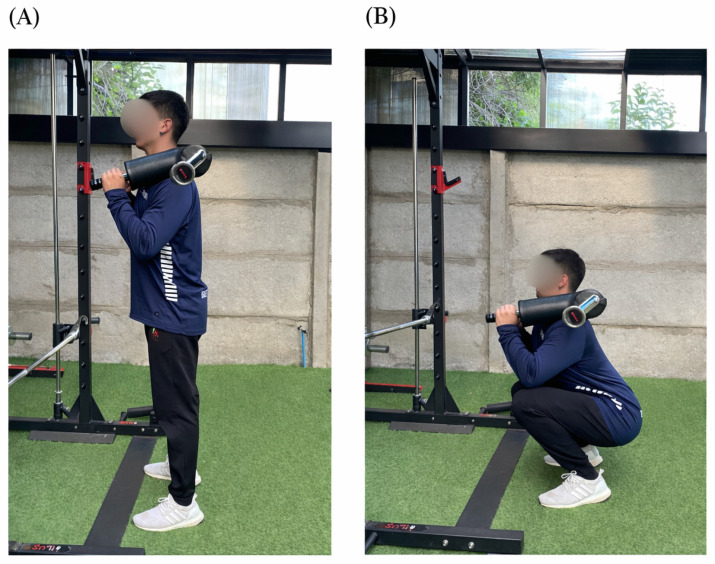
Safety bar squat. (**A**) Standing position prior to the descent. (**B**) Bottom position of the squat.

During the first session, participants provided informed consent, underwent anthropometric assessments, and completed a familiarization protocol with the SSB (Figure 2), as none had prior experience with this implement. Participants were already familiar with the traditional barbell squat through their regular resistance training practice. In the second session, the 1RM was assessed using either the SSB or TRAD, with the order randomized. The third session consisted of the evaluation protocol with the same barbell, performed at 85% 1RM for five repetitions, followed—after a three-minute rest interval—by 65% 1RM for twelve repetitions. This testing sequence was adapted from the protocol described by Vantrease et al. [15] to maintain methodological consistency with previous studies examining velocity-based outcomes during squat exercise. In the fourth session, participants completed the 1RM test with the alternate barbell. Finally, the fifth session replicated the evaluation protocol with the alternate barbell under identical conditions. During all squat trials, participants were instructed to reach a consistent depth corresponding to the thighs at least parallel to the ground. All repetitions were visually supervised by the research team to ensure consistent technique across sessions and barbell conditions.

### 2.7. Statistical Analysis

Statistical analyses were performed using GraphPad Prism (version 9.X; GraphPad Software, San Diego, CA, USA). Descriptive statistics are presented as means, standard deviations, and 95% confidence intervals. A two-way repeated-measures ANOVA (Barbell × load) was used to compare conditions. When significant main or interaction effects were detected, Tukey’s post hoc test was applied for multiple comparisons. Cohen’s effect size was calculated for multiple comparisons to quantify the magnitude of the differences between conditions. Effect sizes (ES) were interpreted according to conventional thresholds as negligible (<0.20), small (0.20–0.49), moderate (0.50–0.79), and large (≥0.80) [23]. Statistical significance was set at *p* < 0.05 for all analyses.

## 3. Results

The descriptive characteristics of the sample are presented in Table 1. Participants had a mean age of 22.48 ± 2.39 years, height 175.03 ± 5.12 cm, body weight of 81.08 ± 10.00 kg, and a BMI of 26.51 ± 3.99 kg/m^2^.

Table 2 presents the descriptive statistics of neuromuscular performance indicators for the TRAD and SSB across both load conditions.

**Figure 3:**
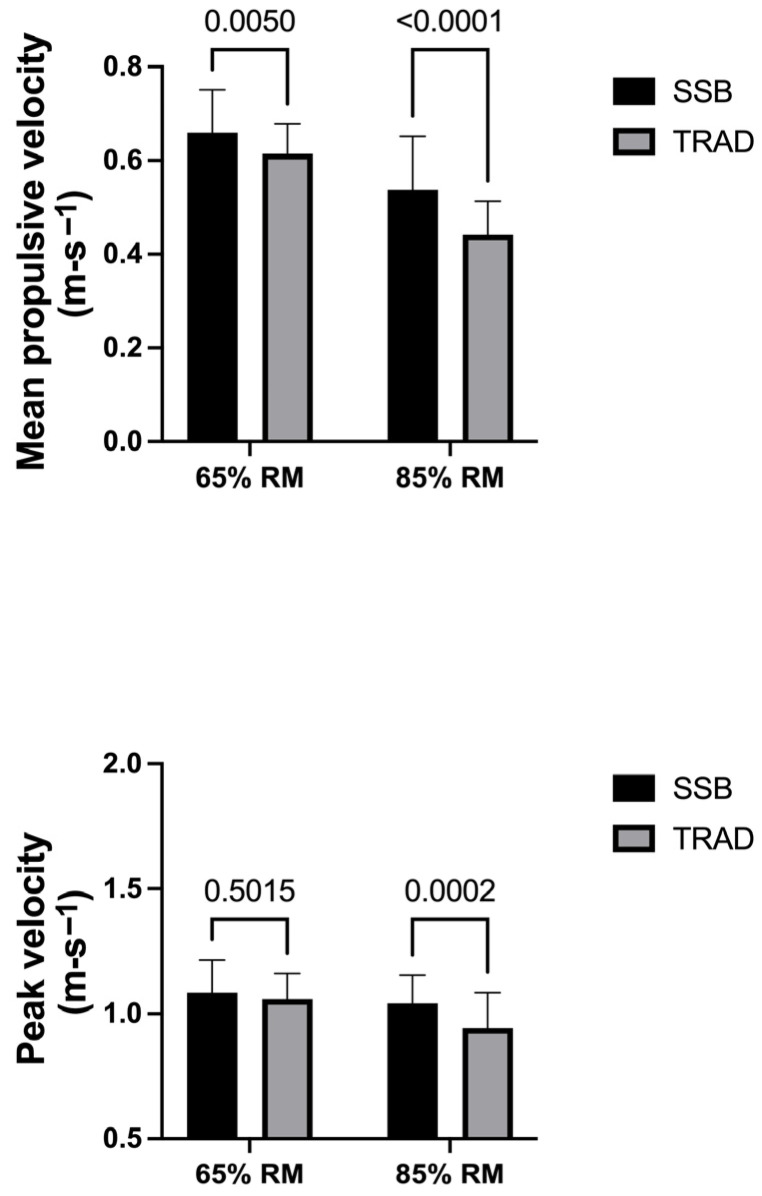
Neuromuscular performance indicators during squat exercise using a safety squat bar (SSB, black bars) and a traditional barbell (TRAD, gray bars) at 65% and 85% of 1RM. Panels show mean propulsive velocity (MPV) and peak velocity (PV).

A two-way repeated-measures ANOVA revealed significant effects for the velocity-based variables. For mean propulsive velocity (MPV), a significant main effect of load was observed (F(1, 18) = 123.4, *p* < 0.0001), indicating lower movement velocities at higher relative loads. A significant main effect of barbell type was also detected (F(1, 18) = 10.94, *p* = 0.0039), with higher MPV values obtained with the safety squat bar compared with the traditional barbell. Additionally, a significant interaction between load and barbell type was found (F(1, 18) = 9.89, *p* = 0.0056), indicating that the magnitude of the velocity differences between barbells varied according to the load condition. For peak velocity (PV), a significant main effect of load was found (F(1, 18) = 20.45, *p* = 0.0003), reflecting lower peak velocities at higher relative loads. A significant main effect of barbell type was also observed (F(1, 18) = 5.615, *p* = 0.0292), with higher PV values recorded with the safety squat bar. In addition, a significant interaction between load and barbell type was detected (F(1, 18) = 7.969, *p* = 0.0113), suggesting that the differences between barbells varied depending on the load level. For velocity loss (VL), a significant main effect of barbell type was observed (F(1, 18) = 7.589, *p* = 0.013), indicating differences between the traditional barbell and the safety squat bar. No significant main effect of load was found (F(1, 18) = 2.207, *p* = 0.1547), and no significant interaction between load and barbell type was observed (F(1, 18) = 0.458, *p* = 0.507). Post hoc comparisons revealed that MPV was significantly higher with the safety squat bar compared with the traditional barbell at both 65% 1RM (*p* = 0.005, d = 0.65) and 85% 1RM (*p* < 0.0001, d = 1.08). For PV, no significant differences were observed between barbell conditions at 65% 1RM (*p* = 0.5015, d = 0.09), whereas significantly higher values were observed with the safety squat bar at 85% 1RM (*p* = 0.0002, d = 1.04). Significant differences between load conditions were also observed for MPV and PV within each barbell condition (all *p* < 0.0001). These results are shown in Figure 3.

## 4. Discussion

The purpose of this study was to compare neuromuscular performance variables during the squat exercise using a traditional barbell (TRAD) and a safety squat bar (SSB). The main findings indicate that the SSB elicited higher movement velocities (MPV and, partially, PV). Additionally, velocity loss (VL) showed a significant main effect of barbell type, although no interaction with load was observed, indicating that differences between bars were consistent across intensities.

The higher MPV values observed with the SSB at both 65% and 85% 1RM align with the notion that barbell design influences movement mechanics and resultant velocity profiles. Previous studies, such as Staheli et al. [16] have also reported significant differences in MPV between SSB and traditional bars, supporting the idea that the forward camber of the SSB and its altered load distribution promote a more upright torso and potentially greater velocity expression during the concentric phase. Although Vantrease et al. [15] also compared TRAD and SSB at similar relative loads, their shorter repetition scheme (three repetitions per intensity) likely limited velocity loss within sets, which may explain the higher velocities reported in their study and the discrepancies with the present findings. The lower velocities observed at 85% compared to 65% 1RM are primarily explained by the load–velocity relationship, whereby higher loads inherently result in lower movement velocities.

Regarding PV, differences emerged only at 85% of 1RM, where the SSB demonstrated significantly greater values than the TRAD, whereas no differences were observed at 65% 1RM. This finding suggests that the potential advantages of the SSB in peak velocity expression may become more pronounced at higher relative loads, possibly due to alterations in squat mechanics associated with bar design. Previous studies have shown that the SSB promotes a more upright trunk position compared to the traditional barbell, which may reduce forward trunk inclination and modify joint moment distribution during the squat [12,14]. The higher velocities observed at lower relative loads are consistent with the established load–velocity relationship, whereby movement velocity decreases as external load increases, reinforcing the relevance of velocity-based monitoring in resistance training contexts [24,25]. Notably, PV did not differ within the SSB across intensities, indicating a relatively stable velocity output despite the increase in load. In contrast, PV declined significantly within the TRAD condition, reinforcing the notion that barbell design may influence how athletes express peak velocity under increasing external loads [15,16].

From a practical standpoint, these findings suggest that barbell selection may influence velocity-based outcomes during squat exercise. The SSB may represent a viable option when the aim is to emphasize velocity expression, whereas the traditional barbell remains a commonly used reference condition for squat performance. However, given the cross-sectional design of the present study and the relatively small sample size, these findings should be interpreted with caution. The testing sequence required participants to perform the 85% 1RM set before the 65% 1RM set. Although this protocol was adapted from previous research comparing TRAD and SSB [15], it may have induced residual fatigue that influenced the velocity outcomes obtained during the subsequent lower-load condition. Therefore, the results obtained at 65% 1RM should be interpreted with caution.

This study is not without limitations. The size and selection of the sample limit the generalizability of the results to other populations (female population or professional athletes). Cross-sectional design prevents conclusions regarding long-term adaptations or training-specific outcomes. In addition, the a priori sample size calculation was based on an effect size derived from a kinetic variable (knee extensor moment), due to the limited availability of velocity-based comparative studies at the time of study design. Therefore, the study may be underpowered for detecting differences in certain kinematic variables. Additionally, although the testing protocol was based on previous literature, the 3 min rest interval between sets may not have allowed for full neuromuscular recovery. Therefore, performance during the 65% 1RM condition may have been influenced by residual fatigue from the preceding 85% 1RM set. Another limitation is that participants had no prior experience with the safety squat bar and completed only one familiarization session before testing. Therefore, part of the observed differences may have been influenced by incomplete motor adaptation to the implement rather than exclusively by its mechanical properties. Future studies should consider longer familiarization periods before baseline testing. Squat depth was supervised visually but not objectively quantified using an external depth-control device or motion-analysis system. Therefore, small between-condition differences in range of motion cannot be completely ruled out. Despite these limitations, the use of validated instruments, standardized procedures, and supervision by qualified professionals strengthens the methodological rigor and reliability of the results.

## 5. Conclusions

In summary, the findings indicate that the SSB elicited higher mean propulsive velocity and, partially, higher peak velocity than the TRAD barbell under the tested conditions. These results suggest that barbell type may influence velocity-based performance during squat exercise. A significant main effect of barbell type was also observed for velocity loss; however, given the absence of interaction effects, this finding should be interpreted with caution. Overall, these results provide preliminary evidence that barbell configuration may influence neuromuscular responses during squat exercise, although further research is needed to confirm these findings under more tightly controlled experimental conditions.

From a practical perspective, these results suggest that barbell selection may influence velocity-based outcomes during squat exercise. The safety squat bar may represent a viable option when the aim is to emphasize movement velocity, whereas the traditional barbell remains a commonly used reference condition for squat performance. However, given the cross-sectional design of the present study, these findings should be interpreted as preliminary, and future longitudinal research is needed to determine whether these differences translate into distinct training adaptations over time.

## Figures and Tables

**Table 1:** Characteristics of the sample.

Variables	Mean ± SD	95% Confidence Interval	
		LL	UL
Age (years)	22.48 ± 2.39	21.33	23.63
Weight (kg)	81.08 ± 10.00	76.26	85.90
Height (cm)	175.03 ± 5.12	172.57	177.49
BMI (kg/m^2^)	26.51 ± 3.99	24.24	27.87
1RM TRAD (kg)	120.23 ± 19.82	110.68	129.78
1RM SSB (kg)	112.20 ± 17.34	103.84	120.56

LL: Lower Limit; UL: Upper Limit; BMI: Body Mass Index; RM: Repetition Maximum.

**Table 2:** Descriptive analysis of Neuromuscular Performance Indicators.

Variables	TRAD			SSB		
	Mean ± SD	95% Confidence Interval		Mean ± SD	95% Confidence Interval	
		LL	UL		LL	UL
MPV 65% at 1RM (m·s^−1^)	0.61 ± 0.06	0.59	0.65	0.66 ± 0.09	0.62	0.70
MPV 85% at 1RM (m·s^−1^)	0.44 ± 0.07	0.41	0.48	0.54 ± 0.11	0.48	0.59
PV 65% at 1RM (m·s^−1^)	1.06 ± 0.10	1.01	1.11	1.05 ± 0.11	1.02	1.15
PV 85% at 1RM (m·s^−1^)	0.94 ± 0.14	0.87	1.01	1.04 ± 0.11	0.99	1.10
VL 65% at 1RM (%)	27.33 ± 6.37	24.26	30.40	22.14 ± 9.18	17.71	26.56
VL 85% at 1RM (%)	25.89 ± 7.48	22.28	29.50	19.06 ± 7.02	15.67	22.44

SD: Standard Deviation; LL: Lower Limit; UL: Upper Limit; MPV: Mean Propulsive Velocity; PV: Peak Velocity; VL: Velocity Loss.

## Data Availability

The data will be made available to interested researchers upon reasonable request.

## Abbreviations

The following abbreviations are used in this manuscript:

VBT	Velocity-Based Training
1RM	One-Repetition Maximum
VL	Velocity Loss
MPV	Mean Propulsive Velocity
PV	Peak Velocity
TRAD	Traditional
SSB	Safety Squat Bar
